# How cell wall complexity influences saccharification efficiency in *Miscanthus sinensis*

**DOI:** 10.1093/jxb/erv183

**Published:** 2015-04-23

**Authors:** Amanda P. De Souza, Claire L. Alvim Kamei, Andres F. Torres, Sivakumar Pattathil, Michael G. Hahn, Luisa M. Trindade, Marcos S. Buckeridge

**Affiliations:** ^1^Laboratory of Plant Physiological Ecology (LAFIECO), Department of Botany, Institute of Biosciences, University of São Paulo, Rua do Matão 277, Sao Paulo, SP, Brazil; ^2^Wageningen UR Plant Breeding, Wageningen University and Research Centre, PO Box 386, 6700 AJ, Wageningen, The Netherlands; ^3^BioEnergy Science Center, Complex Carbohydrate Research Center, The University of Georgia, 315 Riverbend Rd, Athens, GA 30602, USA

**Keywords:** Bioenergy, cell wall polysaccharides, lignin, recalcitrance, saccharification.

## Abstract

The production of bioenergy from grasses has been developing quickly during the last decade, with *Miscanthus* being among the most important choices for production of bioethanol. However, one of the key barriers to producing bioethanol is the lack of information about cell wall structure. Cell walls are thought to display compositional differences that lead to emergence of a very high level of complexity, resulting in great diversity in cell wall architectures. In this work, a set of different techniques was used to access the complexity of cell walls of different genotypes of *Miscanthus sinensis* in order to understand how they interfere with saccharification efficiency. Three genotypes of *M. sinensis* displaying different patterns of correlation between lignin content and saccharification efficiency were subjected to cell wall analysis by quantitative/qualitative analytical techniques such as monosaccharide composition, oligosaccharide profiling, and glycome profiling. When saccharification efficiency was correlated negatively with lignin, the structural features of arabinoxylan and xyloglucan were found to contribute positively to hydrolysis. In the absence of such correlation, different types of pectins, and some mannans contributed to saccharification efficiency. Different genotypes of *M. sinensis* were shown to display distinct interactions among their cell wall components, which seem to influence cell wall hydrolysis.

## Highlight

The manner in which lignin is linked to polysaccharides and the polysaccharide–polysaccharide interactions within cell walls of *Miscanthus sinensis* are associated with recalcitrance to hydrolysis.

## Introduction

The increase in the world’s population and economic activities has resulted in a rising demand for energy (Krausmann *et al.*, 2013). Currently, most of the energy used in the world is derived from fossil fuel resources, which also represent the main source of CO_2_ emissions (Charles *et al.*, 2007; Caspeta *et al.*, 2013). In this context, second-generation bioethanol constitutes a promising renewable alternative to replace oil usage and to increase the world supply of energy (Wyman, 2008; Buckeridge *et al.*, 2010; Magrin *et al.*, 2014).

Second-generation bioethanol production is based on plant biomass, and different species are being considered as promising bioenergy feedstocks (Sims *et al.*, 2007; Buckeridge and Goldman, 2011; van der Weijde *et al.*, 2013; De Souza *et al.*, 2014). *Miscanthus* is among these species since it has a high potential as bioenergy feedstock, especially in the USA, Canada, Europe, and Asia. Important features of *Miscanthus* include high productivity, cold tolerance, and capacity to grow in saline soils (Lewandowski *et al.*, 2003; Heaton *et al.*, 2008; Smeets *et al.*, 2009). Although the genus *Miscanthus* comprises ~17 species (Greef and Deuter, 1993), only the sterile triploid hybrid *Miscanthus*×*giganteus* is currently commercialized (van der Weijde *et al.*, 2013). Due to its sterility, *M.*×*giganteus* lacks genetic variation, which limits breeding for tolerance to environmental stresses and for traits related to biomass quality and bioethanol conversion (Zhao *et al.*, 2014). In fact, besides displaying high yield and low-input requirements for water, nutrients, and pesticides, an ideal bioethanol feedstock should guarantee an efficient and cost-effective conversion of biomass into fermentable monosaccharides (De Souza *et al.*, 2014). The fact that two out of the three subgenomes in *M.×giganteus* originate from *M. sinensis*, and the existence of large genetic diversity in the latter make *M. sinensis* an obvious breeding target to produce new varieties with improved biomass quality (Clifton-Brown *et al.*, 2008; van der Weijde *et al.*, 2013).

The major challenge in converting plant biomass into fermentable sugars to produce second-generation bioethanol is related to the intrinsic cell wall recalcitrance to hydrolysis (Himmel *et al.*, 2007). This recalcitrance is commonly associated with lignin content (Grabber, 2005; Lygin *et al.*, 2011; Vanholme *et al.*, 2012). Indeed, many authors have demonstrated that a decrease in lignin content and/or changes in lignin composition can improve biomass saccharification efficiency (Chen and Dixon 2007; Simmons *et al.*, 2010; Jung *et al.*, 2012; Acker *et al.*, 2014). However, it has been shown that lignin is not the only cell wall component that influences enzymatic saccharification (Ray *et al.*, 2012; Torres *et al.*, 2013; Costa *et al.*, 2014; Marriot *et al.*, 2014; Penning *et al.*, 2014; Torres *et al.*, 2014). Also, it has been suggested that the composition and arrangement of cell wall constituents (Pauly and Keegstra, 2008; Harris *et al.*, 2009; Abramson *et al.*, 2010; Le Ngoc Huyen *et al.*, 2010; De Martini *et al.*, 2014; Torres *et al.*, 2014), as well as the fine structure (i.e. the distribution of sugar and/or non-sugar branching substituents in the main chain) of the polysaccharides (Buckeridge and De Souza, 2014), interfere with cell wall hydrolysis and therefore contribute to recalcitrance.

*Miscanthus* has cell walls typical of grasses (Le Ngoc Huyen *et al.*, 2010; Lygin *et al.*, 2011). Specifically, analysis of a collection of 102 accessions of *M. sinensis* showed that the cell walls are composed of 30–44% cellulose, 29–42% hemicellulose, and 7–21% lignin (Zhao *et al.*, 2014). The most abundant cell wall monosaccharides in *Miscanthus* are glucose, xylose, and arabinose, the latter two originating primarily from arabinoxylan, which is the main hemicellulose of this plant (Le Ngoc Huyen *et al.*, 2010; Lygin *et al.*, 2011; Domon *et al.*, 2013). Another hemicellulosic polysaccharide present in *Miscanthus* cell walls is the mixed linkage β-glucan that has been reported to account for 1.5% of the wall (Domon *et al.*, 2013).

Xu *et al.* (2012) demonstrated that hemicelluloses are positively correlated to the hexose yield upon saccharification of *Miscanthus* after pre-treatment with alkali and acid, whereas cellulose and lignin were negatively correlated. These authors propose the use of genetic engineering to increase hemicellulose in *Miscanthus*, but did not examine which hemicellulose would be more beneficial to saccharification of *Miscanthus* biomass.

Although the composition of *Miscanthus* cell walls has been previously described and some authors have correlated the amount of hemicelluloses to a higher saccharification efficiency, most reports take into consideration the general classes of polymers in the wall and seldom examine hemicelluloses or pectin polymers in detail. This could be important to help in explaining saccharification, as proposed by De Souza *et al.* (2013) for sugarcane walls, which are compositionally close to those of *Miscanthus*. Furthermore, the analysis of the fine structure of hemicellulosic polymers is also important due to the fact that these polymers are thought to hold a significant proportion of the complexity present in cell walls, which is partially due to the combination of monosaccharides and decorations with methyl, acetyl, and phenylpropanoids (Buckeridge and De Souza, 2014).

Considering that more in-depth analyses of the complexity in *Miscanthus* cell walls have not yet been assessed, in the present work a series of genotypes of *M. sinensis* were analysed in order to evaluate the importance of the fine structure of the polysaccharides present in their cell walls for recalcitrance/saccharification of biomass. Combining different cell wall analytical techniques, the fine structure of *Miscanthus* cell wall polysaccharides is first described, and subsequently the role of pectins and hemicelluloses in hydrolysis/recalcitrance is discussed. It was found that when lignin is not correlated with saccharification efficiency, pectin and mannan epitopes detected in the glycome profiles seem to favour hydrolysis, whereas recalcitrance is mostly explained by certain epitopes of xyloglucan and arabinoxylan. However, when lignin is negatively correlated with saccharification efficiency, different epitopes of arabinoxylan, xyloglucan, and pectins contributed to hydrolysis, whereas another type of pectins, probably associated with lignin, contributed to recalcitrance. This work unravels part of the cell wall complexity involved in hydrolysis, possibly helping to design future strategies for more efficient cell wall disassembly for bioenergy production.

## Materials and methods

### Plant material

Seven *M. sinensis* genotypes from the *Miscanthus* collection at Wageningen University and Research Centre (WUR) were selected based on their contrasting cell wall composition. Stems from five plants were harvested 4 weeks after the onset of heading, to ensure the analysis on full-grown stems. The collected samples were chopped and air-dried at 70 °C for 48h, and were subsequently ground to a fine powder using a ball mill (Retsch, Haan, Germany). The cell walls of the seven genotypes were analysed for lignin, hemicelluloses, and cellulose contents, as well as for cell wall monosaccharide composition. Three genotypes with contrasting compositions for cell wall polysaccharides were chosen for more in-depth analyses using cell wall fractionation and glycome profiling techniques.

Composition analyses of classes of polymers: cellulose, hemicellulose, and lignin

Neutral detergent fibre (NDF) and acid detergent fibre (ADF) components were determined as described by Torres *et al.* (2013). The lignin content was quantified by the acetyl bromide method, which uses the cell wall residue obtained from the NDF treatment to measure the extractable soluble lignin (AcBrLIG%). The determination of cellulose and hemicelluloses contents was based on the results obtained from the NDF, ADF, and lignin methods. The NDF residue comprises hemicelluloses, cellulose, and lignin, whereas the ADF residue consists of cellulose and lignin. Thus, the content of cellulose was measured by subtracting the AcBrLIG% from the ADF% values, while the content of hemicelluloses was determined by subtracting the ADF% from the NDF%. Three technical replicates were performed per genotype, and the lignin content was calculated using the formula given below and the extinction coefficient mentioned by Fukushima and Kerley (2011).

$$X=\frac{\left(Y-0.009\right)}{23.077}$$

Where, X=lignin concentration (mg/ml); Y=optical density reading; 0.009=mean intercept value; and 23.077=mean extinction coefficient obtained from Fukushima and Kerley (2011).

### Cell wall preparation

A 500mg aliquot of each sample was subjected to six consecutives extractions with 25ml of 80% (v/v) ethanol at 80 °C for 20min. Each extraction was followed by centrifugation (10min at 8500 *g*) and the supernatant was discarded to remove the soluble sugars. The samples were subjected to an overnight extraction with 20ml of 90% (v/v) dimethylsulphoxide (DMSO) with continuous stirring followed by centrifugation to remove starch. The remaining material, consisting of cell walls, was washed with distilled water and dried at 60 ºC for 24h.

### Cell wall fractionation

In order to solubilize polysaccharides, the cell walls were subjected to consecutive extractions with 0.5M ammonium oxalate (pH 7.0), sodium chlorite in 0.3% (v/v) acetic acid, and 0.1, 1.0, and 4.0M NaOH as previously described in De Souza *et al.* (2013). All solubilized extracts were neutralized, dialysed to remove salts, and freeze-dried. The extract yields were obtained gravimetrically.

### Monosaccharide composition

Each cell wall extract and intact cell wall sample was hydrolysed with 2M trifluoroacetic acid (TFA) for 1h at 100 °C. The acid was evaporated under vacuum and the monosaccharides were resuspended in 2ml of ultra-purified water. Monosaccharide profiles were analysed by high-performance anion exchange chromatography with pulsed amperometric detection (HPAEC-PAD) on a CarboPac SA10 column (DX-500 system, Dionex^®^) using a mixture of 99.2% water and 0.8% (v/v) 150mM NaOH as eluent (1ml min^–1^). The monosaccharides were detected with a post-column addition of base with 500mM NaOH (1ml min^–1^).

### Oligosaccharide profiling

The oligosaccharide profiles were obtained by incubation of 1% (w/v) of each extract or intact cell wall sample with selected endoglycanases in 50mM sodium acetate buffer, pH 5.0 at 30 °C for 24h. The substrates were incubated with xylanase (Sigma^®^, cat. X2753), lichenase (Megazyme^®^, cat. E-LICH), or GH12 xyloglucan endoglucanase (XEG) according De Souza *et al.* (2013) in order to access the fine structures of arabinoxylan, β-glucan, and xyloglucan, respectively. The oligosaccharides produced were analysed by HPAEC-PAD on a CarboPac PA-100 (ICS-3000 system, Dionex^®^) using 88mM NaOH and 200mM sodium acetate as eluent (0.9ml min^–1^) for 45min.

### Glycome profiling

Glycome profiling analysis of the cell wall extracts was performed using an enzyme-linked immunosorbent assay (ELISA) test (Pattathil *et al.*, 2012) with a comprehensive library of cell wall glycan-directed monoclonal antibodies (Pattathil *et al.*, 2010).

### Saccharification

Biomass samples (50mg) were thermochemically pre-treated at a 1.7% solids loading in 0.33% (w/v) sulphuric acid for 60min at 110 °C. Reactions were carried out on a heating block using 15ml Kimax glass tubes sealed with teflon-lined plastic caps. Following thermochemical treatment, liquors were removed by suction using a mechanical pipette.

Enzymatic saccharification of pre-treated samples was performed following a modified version of the Laboratory Analytical Procedure-009 from the National Renewable Energy Laboratory (NREL). Briefly, pre-treated samples were treated with 25 μl of an Accelerase 1500 cellulolytic enzyme cocktail (Genencor BV, Leiden, The Netherlands) in 10ml of 0.1M citrate buffer. The enzyme load provided 50 filter paper units (FPU) of cellulase per gram of cellulose. Samples were subsequently incubated at 50 °C in an Innova 42 air incubator (New Brunswick Scientific, Enfield, CT, USA) at 200rpm for 24h. Enzymatic saccharification liquors were analysed for glucose content using a Boehringer Mannheim d-Glucose kit (Boehringer Mannheim, Indianapolis, IN, USA). The colorimetric assay was adapted to a 96 microtitre plate format, and spectrophotometric reads were made using a Bio-Rad 550 Micro-plate Reader (Bio-Rad, Richmond, CA, USA). For all samples, glucose content was expressed as both the amount of glucose released from dry biomass (Glc-Rel) and the percentage of total cell wall glucose released upon enzymatic saccharification (Glc-Con).

### Data analysis

Differences among the genotypes (*n*=5) were analysed using a General Linear Model (GLM) followed by Tukey test with significance *P*<0.05.

In order to evaluate which cell wall components are likely to be more relevant to saccharification and to recalcitrance, a principal component analysis (PCA) was performed. The PCA considered only variables that were statistically different among genotypes, according to the results of the GLM analysis. In order to configure the glycome profiling data into a form that is suitable for PCA, the ELISA signals for antibodies that bound to a given class of polysaccharide were averaged according to the colour code displayed in Supplementary Table S2 available at *JXB* online. PCA loadings were used to calculate the percentage contribution of each cell wall variable.

All data analysis was performed using the software Minitab^®^, version 14.1.

## Results

### Cell wall general composition and the choice of genotypes for polysaccharide analyses

The analysis of the general biomass composition of the seven *M. sinensis* genotypes showed different ranges of lignin, hemicelluloses, and cellulose contents within the group of genotypes analysed (Supplementary Fig. S1 at *JXB* online). As the goal of the present work was to analyse the possible role of polysaccharides in recalcitrance of *M. sinensis* cell walls, the three genotypes with the lowest lignin content (H0116, H0120, and H0198) (Supplementary Fig. S1A) were chosen for saccharification and more in-depth analyses of cell wall polysaccharides. Within this group of three genotypes, two different patterns were observed: (i) one genotype (H0198) with lower and two (H0116 and H0120) with higher hemicellulose content; and (ii) two genotypes (H0120 and H0198) with lower content and one (H0116) with higher cellulose content (Supplementary Figs. S1B, C). These three genotypes also showed some significant differences in monosaccharide compositions of their cell walls (Supplementary Table S1), which led to differences in their hexose:pentose ratio (H:P) (Supplementary Fig. S2). The H0120 genotype showed a lower H:P, while the H0116 genotype had a higher H:P.

### Analyses of cell wall components of three genotypes of *Miscanthus sinensis*

**Fig. 1.**
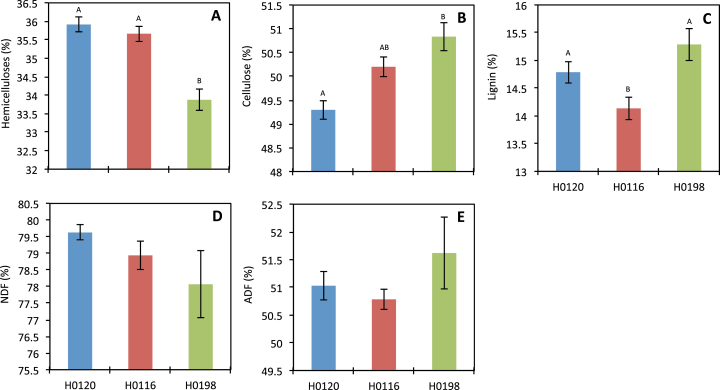
Contents of (A) hemicelluloses, (B) cellulose, (C) lignin, (D) neutral detergent fibre (NDF), and (E) acid detergent fibre (ADF) components of three different genotypes (H0120, H0116, and H0198) of *Miscanthus sinensis*. Different letters indicate statistically significant differences (*n*=5). (This figure is available in colour at *JXB* online.)

**Fig. 2.**
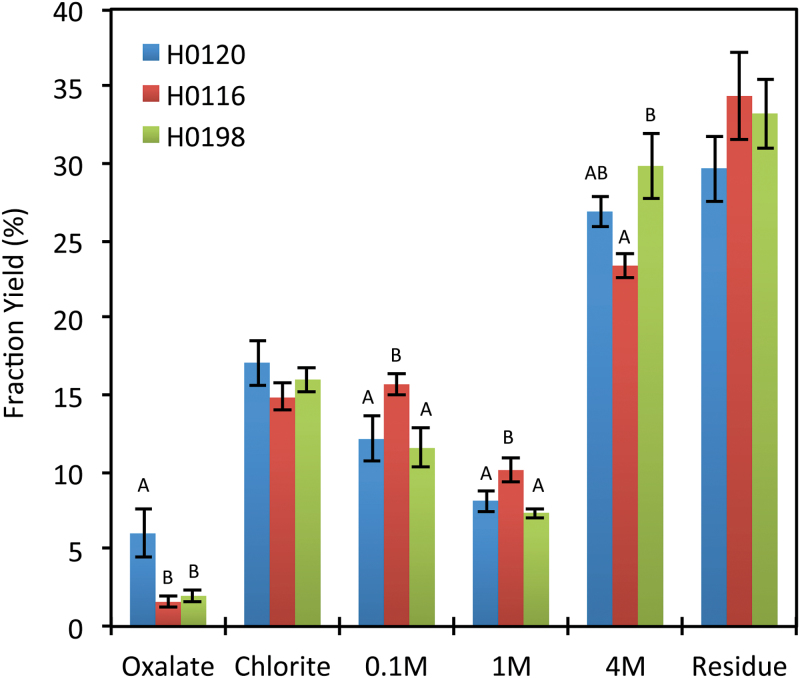
Cell wall extracts yield (%) of three different genotypes (H0120, H0116, and H0198) of *Miscanthus sinensis*. Different letters indicate statistically significant differences (*n*=5). (This figure is available in colour at *JXB* online.)

Genotype H0198 had a lower hemicelluloses content (5.5%) in its cell walls than did the other two genotypes, H0120 and H0116 (Fig. 1A). This same trend was observed in wall fraction yields for H0198, where the 0.1M and 1M NaOH extracts yielded less material, although the difference between H0198 and H0120 was not significant (Fig. 2). The H0120 genotype had ~2.3% lower cellulose content than H0198 (Fig. 1B). Similarly, the yield of residue from the fractionation procedure of this genotype showed a tendency to be lower than H0116 and H0198. The ammonium oxalate extract of H0120 was significantly higher (Fig. 2), suggesting that this genotype is composed of a higher proportion of soluble polysaccharides than H0116 and H0198.

The lignin content was 7.5% and 4.4% lower in the H0116 genotype compared with H0198 and H0120, respectively (Fig. 1C). No significant differences among genotypes were observed in NDF% and ADF% (Fig. 1D, E).

The analysis of monosaccharides by HPAEC/PAD revealed significant differences among the three genotypes associated with proportions of arabinose, galactose, glucose, rhamnose, and xylose in different cell wall fractions. Fucose and mannose did not show any statistical differences among the three genotypes (Table 1).

**Table 1.** M*onosaccharides (%) from the cell wall extracts of three different genotypes (H0120, H0116,* and H0198) of Miscanthus sinensis Statistically significant differences are shown in bold (*n*=5).

Cell wall extracts	Genotypes	Monosaccharides (%)						
		Arabinose	Fucose	Galactose	Glucose	Mannose	Rhamnose	Xylose
Ammonium oxalate	H0120	**11.11±0.65 a**	0.11±0.02	2.76±1.08	**7.31±2.33 a**	0.31±0.20	**0.02±0.01 a**	**78.37±4.23 a**
	H0116	**9.03±1.34 a,b**	0.06±0.01	3.91±1.12	**39.57±4.64 b**	0.72±0.28	**0.26±0.09 b**	**46.45±4.66 b**
	H0198	**6.57±1.31 b**	0.11±0.07	2.37±0.44	**42.93±11.74 b**	0.35±0.05	**0.12±0.04 c**	**56.50±5.16 b**
Sodium chorite	H0120	12.36±0.21	0.11±0.03	1.84±0.11 a	4.08±0.34	0.16±0.03	0.08±0.02	81.14±0.30
	H0116	11.82±0.38	0.08±0.02	1.63±0.04 a	8.16±1.57	0.08±0.05	0.06±0.01	78.17±1.35
	H0198	10.98±0.78	0.07±0.02	1.35±0.10 b	8.55±2.41	0.17±0.01	0.04±0.03	78.84±1.79
0.1M NaOH	H0120	13.01±0.47	0.10±0.03	1.37±0.04	**1.06±0.08 a,b**	0.04±0.01	0.04±0.02	**84.38±0.44 a,b**
	H0116	13.53±0.59	0.13±0.01	1.29±0.08	**1.73±0.34 a**	0.09±0.03	0.10±0.04	**83.13±0.45 b**
	H0198	12.40±0.50	0.13±0.01	1.30±0.03	**0.77±0.09 b**	0.05±0.01	0.07±0.03	**85.30±0.56 a**
1M NaOH	H0120	7.41±0.44	0.13±0.03	0.40±0.04	0.96±0.10	0.11±0.05	**0.06±0.02 a**	90.93±0.37
	H0116	7.35±0.18	0.11±0.02	0.39±0.02	1.10±0.24	0.02±0.01	**0.05±0.00 a**	90.99±0.20
	H0198	7.56±0.29	0.14±0.02	0.33±0.03	0.71±0.09	0.12±0.03	**0.11±0.02 b**	91.03±0.41
4M NaOH	H0120	6.43±0.22	0.03±0.01	**0.89±0.05 a**	4.17±0.91	0.25±0.03	0.04±0.01	88.20±0.81
	H0116	6.62±0.08	0.03±0.01	**0.91±0.04 a**	4.40±0.75	0.27±0.04	0.02±0.01	87.75±0.75
	H0198	6.25±0.15	0.03±0.00	**0.62±0.03 b**	3.76±0.23	0.22±0.02	0.02±0.01	89.10±0.39
Residue	H0120	2.69±0.72	0.56±0.18	0.84±0.32	83.12±4.14	0.41±0.13	ND	12.38±3.87
	H0116	2.79±1.17	0.67±0.12	1.08±0.67	86.21±4.35	0.37±0.02	ND	8.81±2.64
	H0198	1.49±0.28	0.53±0.15	0.44±0.13	89.81±2.24	0.34±0.11	ND	7.34±1.85

Compared with H0120 and H0116, H0198 has a lower proportion of arabinose in the ammonium oxalate extract, a lower proportion of galactose in the sodium chlorite and 4M NaOH extracts, a lower proportion of glucose and a higher proportion of xylose in the 0.1M NaOH extract (Table 1).H0120 has higher percentages of arabinose and xylose in the ammonium oxalate-solubilized fraction compared with the other two genotypes, which is probably associated with the extraction of a more soluble arabinoxylan-like polymer. Concomitantly, H0120 has a much lower proportion of glucose in this same cell wall fraction (~80% lower than that in H0116 and H0198). H0116 has higher percentage of rhamnose in the ammonium oxalate extract and a lower proportion of xylose in this same cell wall fraction, suggesting that more pectin is being extracted in this fraction for this genotype (Table 1).

### Oligosaccharide profiling

**Fig. 3.**
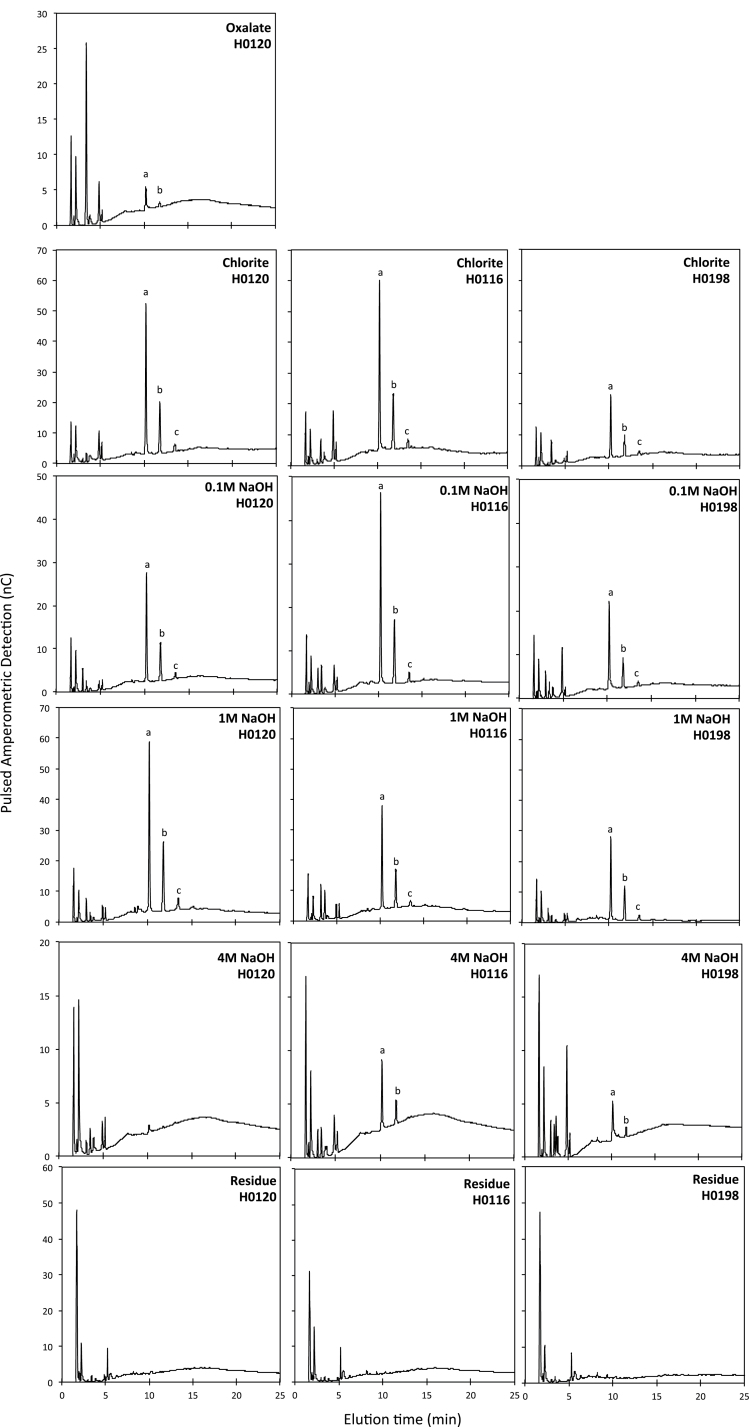
Oligosaccharide profiles obtained using lichenase (for detection of β-glucan) of cell wall fractions of stems of three different genotypes (H0120, H0116, and H0198) of *Miscanthus sinensis*. (a) Trisaccharides, (b) tetrasaccharides, and (c) pentasaccharide. Missing chromatograms from the ammonium oxalate extract of the H0116 and H0198 genotypes are due to the low amount of sample in this extract. Data on chromatograms are the average of three biological replicates.

The oligosaccharides profiles obtained after digestion with lichenase showed that the mixed linkage β-glucan was extracted by sodium chlorite and sodium hydroxide (0.1, 1, and 4M) during the preparation of cell wallextracts of all three genotypes, with the exception of the 4M NaOH extract from H0120, in which no peaks associated with tri- or tetrasaccharides were observed (Fig. 3). No evidence for the presence of β-glucan in the residue (cellulose) remaining after the last base extraction was found by means of the oligosaccharide analyses. However, evidence that some β-glucan might be present in the residue was obtained in the glycome profiles (see below).

The H0198 genotype yielded peaks related to tri- and tetrasaccharides, with lower intensity in all analysed fractions, suggesting that this genotype contains relatively less β-glucan. This is consistent with the lower content of glucose in the ammonium oxalate and 0.1M NaOH extracts found in the H0198 genotype (Table 1), but contradictory to what has been observed in the glycome profiles. On the other hand, both H0120 and H0116 seem to have similar amounts of β-glucan, although the proportions among the extracts are different. H0116 yielded greater peaks in the 0.1M NaOH extract, whereas the higher peaks in H0120 were observed in the 1M NaOH-solubilized fraction (Fig. 3), denoting differences in polysaccharide extractability and/or binding to other polymers between these two genotypes.

Cell wall extracts were also treated with endo-β-xylanase, an enzyme that cleaves β-1,4 linkages in the unbranched portions of the arabinoxylan main chain. The oligosaccharide profiles showed no significant differences among genotypes, suggesting that arabinoxylan fine structures could be quite similar for the three genotypes (Supplementary Fig. S3 at *JXB* online). Although in low proportions compared with β-glucan and arabinoxylan, xyloglucan oligosaccharides released after digestion with XEG (GH12) showed slight differences among genotypes. Xyloglucan was lower in the sodium chlorite and 4M NaOH extracts in the H0120 genotype. The H0116 genotype yielded higher peaks related to xyloglucan in the 4M NaOH extract, with concomitant lower peaks in the 1M NaOH extract, which suggests the presence of relatively more xyloglucans possibly bound to cellulose in this genotype. Despite those differences, it was not possible to identify the types of oligosaccharides released by XEG (Supplementary Fig. S4).

### Glycome profiling

**Fig. 4.**
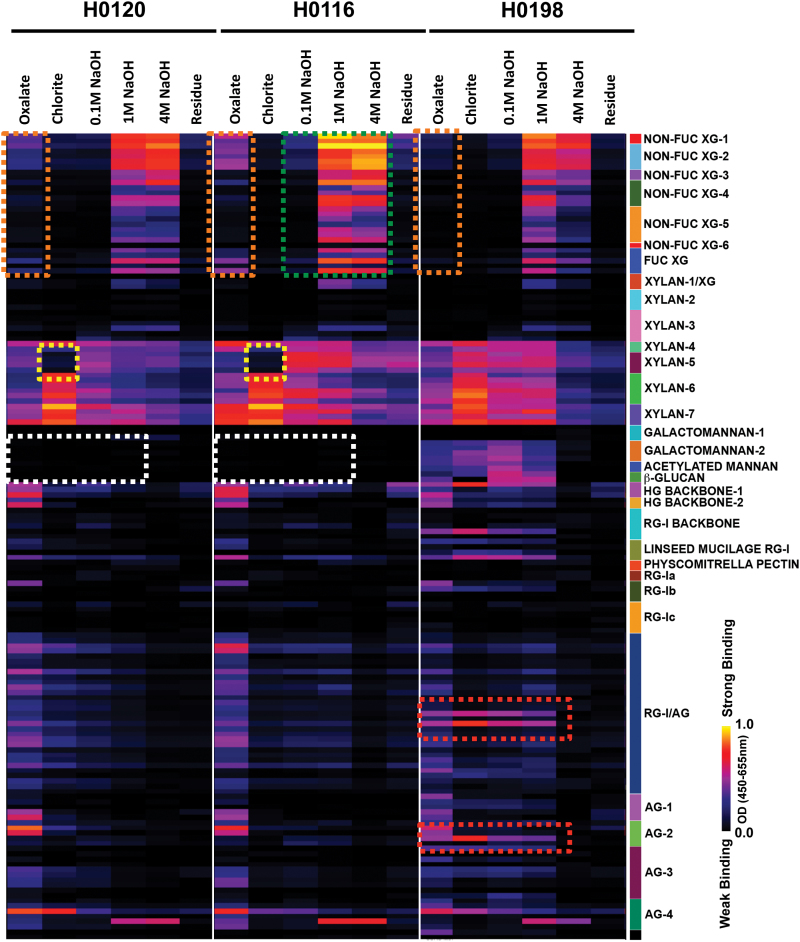
Glycome profiles of each cell wall extract of three different genotypes (H0120, H0116, and H0198) of *Miscanthus sinensis*. On the right, the groups of antibodies used are shown, identified according to the polysaccharides predominantly recognized by each group. Coloured boxes indicate the main statistically significant differences among genotypes (*n*=5). See Supplementary Table S2 at *JXB* online for additional details about the individual antibodies used and statistics.

The glycome profiles of cell wall extracts showed significant differences in the pattern and extractability of diverse glycan epitopes present in these three genotypes (Fig. 4; Supplementary Table S2 at *JXB* online). Specifically, the overall profiles of H0120 and H0116 are quite similar, and clearly distinct from the overall profile for H0198. For example, epitopes recognized by the xylan-5 clade of antibodies are absent in the sodium chlorite extract of the H0120 and H0116 genotypes, but present in H0198 (Fig. 4, yellow boxes). In contrast, the H0120 and H0116 genotypes appear to lack any mannan or β-glucan epitopes, while both sets of epitopes are clearly abundant in extracts of the H0198 genotype (Fig. 4, white boxes). The H0120 and H0116 genotypes had significantly higher levels of xyloglucan epitopes released in the oxalate extract than did the H0198 genotype (Fig. 4, orange boxes). H0116 appeared to contain higher levels of xyloglucan epitopes overall than did the other two genotypes (Fig. 4, green box). A few arabinogalactan-directed antibodies showed higher binding to wall extracts of H0198 [two antibodies in the middle of the rhamnogalacturonan I (RG-I)/arabinogalactan (AG) group and one in the AG-2 group] than were observed in extracts from the other two genotypes (Fig. 4, red boxes).

### Saccharification

**Fig. 5.**
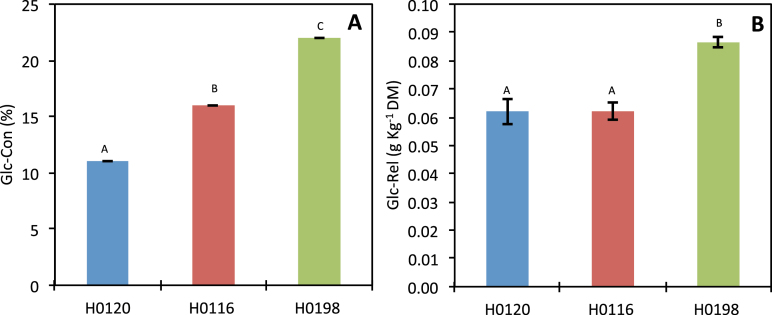
Saccharification of three different genotypes (H0120, H0116, and H0198) of *Miscanthus sinensis*. (A) Percentage of the total cell wall glucose released after enzymatic hydrolysis (Glc-Con); (B) amount of glucose released based on dry biomass (Glc-Rel). (This figure is available in colour at *JXB* online.)

The saccharification efficiency of stems differed significantly among genotypes (Fig. 5). The H0198 genotype yielded the highest percentage of glucose conversion (22%) from the total available cell wall glucose (Fig. 5A). On the other hand, H0120 was the genotype that showed the lowest values of glucose conversion (12%). The same pattern of saccharification efficiency was observed when the data were expressed by the amount of glucose released from 1g of dry biomass (Fig. 5B).

**Fig. 6.**
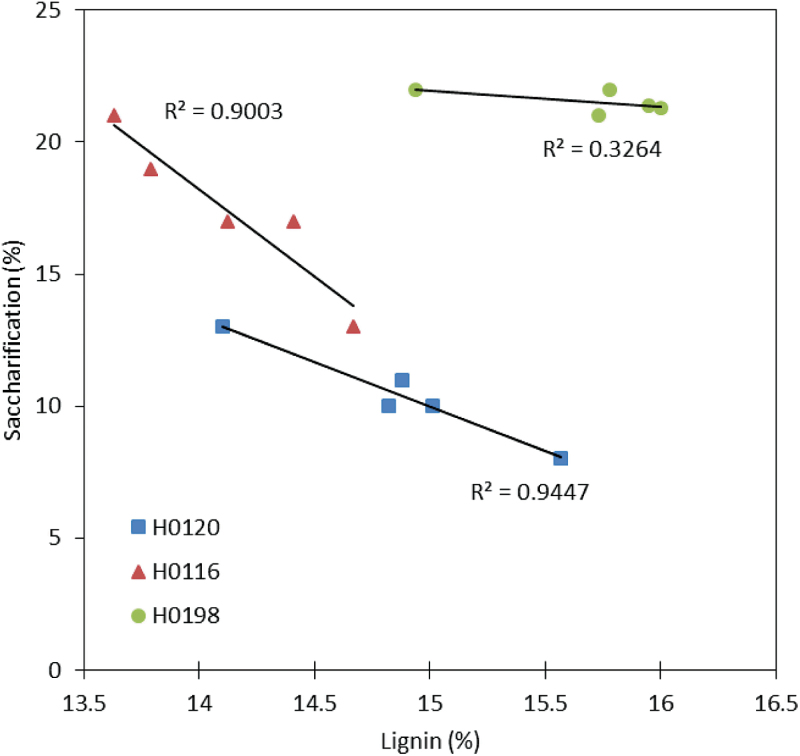
Linear regression between lignin content (%) and saccharification efficiency (%) of three different genotypes (H0120, H0116, and H0198) of *Miscanthus sinensis*. (This figure is available in colour at *JXB* online.)

Lignin affected saccharification efficiency of cell walls of the three genotypes differently (Fig. 6). Whereas H0116 and H0120 showed a clear negative correlation between saccharification efficiency and the percentage of lignin, the same was not observed for the H0198 genotype. This raised the question of whether in certain cases other factors such as polysaccharide composition might interfere more strongly with saccharification efficiency.

**Fig. 7.**
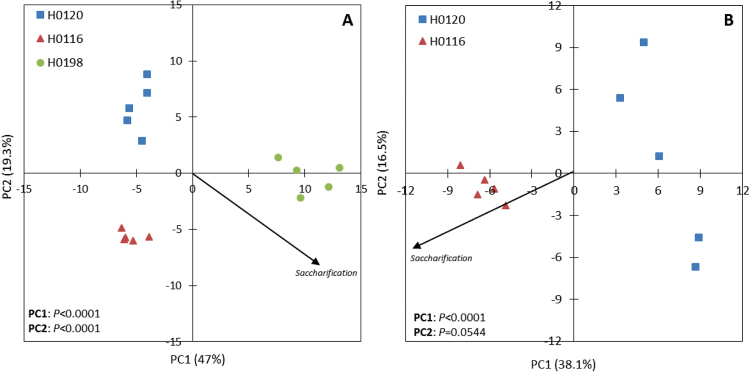
Principal component analysis (PCA) of the cell wall variables that showed statistically significant differences according to GLM results (*P*<0.05). (A) Data from the three genotypes showing the saccharification vector separating mainly the H0198 genotype from the others at PC1. (B) Data from H0120 and H0116 genotypes (both inversely correlated with lignin content) showing the saccharification vector at PC1. *P*-values indicate statistically significant differences of PCs. (This figure is available in colour at *JXB* online.)

In order to check this hypothesis, a PCA considering all cell wall components found to be statistically significant among genotypes was performed. The PCA revealed clear differences among the three genotypes, with PC1 contributing with 47% of the total variation (Fig. 7A). PC1 distinguished H0198 from the other two genotypes. At the same time, this principal component also displays the highest contribution for saccharification efficiency (Supplementary Table S3 at *JXB* online). This makes PC1 the most adequate component to distinguish features of cell wall polysaccharides correlated to saccharification or recalcitrance. Therefore, the percentages contributions to saccharification or recalcitrance (Table 2) were calculated from PC1 loadings (Supplementary Table S3). The variables chosen as the highest influences on either feature were the ones in the top 50% of the PC1. Among these selected variables, the 25 favouring saccharification efficiency were monoclonal antibodies detecting epitopes in polysaccharides belonging to the classes of pectins (~28%), mannans (16%), β-glucan (~4%), and xylan-5 (2%) (Table 2A). These variables are associated mainly with the 0.1M NaOH- and sodium chlorite-solubilized cell wall fractions. Twenty variables were shown to be associated with higher recalcitrance, corresponding to different variables and epitopes, including: xyloglucan (~26%), xylans (~13%), pectins (~8%), and galactose in the 4M NaOH extract (2.8%), and the percentage of hemicelluloses (2.5%) (Table 2B). The xylans and xyloglucans related to recalcitrance are associated with the ammonium oxalate- and 4M NaOH-solubilized wall fractions. Additionally, some pectins present in the ammonium oxalate extract and the residue seem to be related to recalcitrance. Although xylans and pectins are also present in the group of variables that favours saccharification efficiency, the cell wall fractions from which these polysaccharides were released were completely different (e.g. pectins released from sodium chlorite, and 0.1M and 1M NaOH cell wall extracts favour saccharification efficiency, while pectins from ammonium oxalate-solubilized and residue wall fractions favour recalcitrance). Moreover, it is possible to observe differences in antibody epitopes related to saccharification efficiency and recalcitrance.

**Table 2.** *Percentage contribution of different cell wall features of* Miscanthus sinensis *to saccharification (A and C) and recalcitrance (B and D) based on the results from principal component analysis* (A, B) Considering the three genotypes, showing the contribution for saccharification and recalcitrance without the interference of lignin content in PC1, (C, D) considering the results from PCA between H0120 and H0116, where lignin content interferes in saccharification in PC1.

Without lignin interference											
(A) Positively correlated to saccharification (favouring saccharification)						(B) Negatively correlated to saccharification (favouring recalcitrance)					
Polysaccharide	Variable	Extract	% from PC1	Total % per polysaccharide		Polysaccharide	Variable	Extract	% from PC1	Total % per polysaccharide	
Pectins	RG1-b	Sodium chlorite	2.05	27.9%		Xyloglucan	NFXG-3	Ammonium oxalate	2.86	25.9%	
	Linseed-MUC		2.03				NFXG-4		2.79		
	RG-1		2.00				NFXG-2		2.74		
	AG-1		1.89				NFXG-1		2.66		
	HG-2	0.1M NaOH	2.08				NFXG-5		2.64		
	RG1-b		2.08				FXG		2.58		
	Linseed-MUC		2.06				NFXG-2	4M NaOH	2.48		
	AG-1		2.06				NFXG-1		2.41		
	AG-3		2.05				FXG		2.43		
	AG-2		1.95				NFXG-3		2.33		
	RG-1		1.90			Xylan	Xylan-3	Ammonium oxalate	2.94	12.9%	
	RG-1	1M NaOH	2.00				Xylan-2		2.30		
	RG1-b		1.90				Xylan-1		2.46		
	AG-3		1.89				Xylan-2	4M NaOH	2.61		
Mannans	GM-2	Ammonium oxalate	2.00	16.0%			Xylan-3		2.56		
	ACMAN		2.01			Pectins	RG1-a	Ammonium oxalate	2.76	7.9%	
	GM-2	Sodium chlorite	2.03				HG-1		2.56		
	ACMAN		2.03				RG1-a	Residue	2.56		
	GM-2	0.1M NaOH	2.08				Galactose (%)	4M NaOH	2.84	2.8%	
	ACMAN		2.10				% Hemicellulose		2.48	2.5%	
	GM-2	1M NaOH	1.87								
	ACMAN		1.87								
β-Glucan	BG	0.1M NaOH	2.01	3.9%							
	BG	1M NaOH	1.87								
Xylan-5	Xylan-5	Sodium chlorite	2.05	2.0%							
With lignin interference											
(C) Positively correlated to saccharification (favouring saccharification)						(D) Negatively correlated to saccharification (favouring recalcitrance)					
Polysaccharide	Variable	Extract	% from PC1		Total % per polysaccharide	Polysaccharide	Variable	Extract	% from PC1		Total % per polysaccharide
Xylan	Xylan-4	Ammonium oxalate	1.84		19.8%	Pectins	AG-1	Sodium chlorite	3.31		28.2%
	Xylan-7		1.79				AG-2		3.75		
	Xylan-5	0.1M NaOH	2.19				AG-3		3.56		
With lignin interference											
(C) Positively correlated to saccharification (favouring saccharification)						(D) Negatively correlated to saccharification (favouring recalcitrance)					
Polysaccharide	Variable	Extract	% from PC1		Total % per polysaccharide	Polysaccharide	Variable	Extract	% from PC1		Total % per polysaccharide
	Xylan-6		2.16				AG-4		3.87		
	Xylan-7		1.91				RG/AG		3.75		
	Xylan-1	1M NaOH	1.95				Linseed-MUC		3.45		
	Xylan-4		2.16				AG-1	4M NaOH	3.22		
	Xylan-5		2.10				HG-2	Residue	3.25		
	Xylan-6		2.06			Xylan	Xylan-1	Ammonium oxalate	2.89		9.6%
	Xylan-1	4M NaOH	1.7				Xylan-1	Sodium chlorite	3.05		
Xyloglucan	FXG	Ammonium oxalate	1.94		16.9%		Xylan-2	4M NaOH	2.89		
	NFXG-2		1.94			β-Glucan	BG	1M NaOH	3.39		6.7%
	FXG	1M NaOH	1.78				BG	Residue	3.33		
	NFXG-1		2.08				Xylose (%)	Ammonium oxalate	3.33		3.3%
	NFXG-2		1.83				Lignin (%)		2.97		3.0%
	NFXG-4		1.81								
	NFXG-5		1.87								
	NFXG-6		1.95								
	NFXG-6	4M NaOH	1.73								
Pectins	Linseed-MUC	0.1M NaOH	1.76		9.3%						
	RG1-b	1M NaOH	2.06								
	HG-1		1.94								
	Linseed-MUC		1.83								
	RG/AG		1.73								
	Glucose (%)	Ammonium oxalate	2.02		2.0%						

When the same type of analysis was carried out comparing the genotypes H0120 and H0116 (Fig. 7B; Supplementary Table S4 at *JXB* online), both displaying the influence of lignin on hydrolysis/recalcitrance (Fig. 6), it was found that different types of xylans, mainly present in the 0.1M NaOH- and oxalate-solubilized wall fractions, favoured hydrolysis (Table 2C), whereas recalcitrance was favoured by the lignin content, with the main polysaccharide fraction affected displaying epitopes of pectins present in the chlorite extract(Table 2D).

## Discussion

Although there are several publications in which the cell walls of *Miscanthus* have been analysed for their composition (Hodgson *et al.*, 2010; Le Ngoc Huyen *et al.*, 2010; Allison *et al.*, 2011; Domon *et al.*, 2013; Zhao *et al.*, 2014), the fine structure of its cell wall polymers has not been assessed. It has already been suggested that the fine structure of polysaccharides could play a role in saccharification efficiency, because it is associated with the motifs that will be available to enzyme action (Buckeridge and De Souza, 2014).

In the present work, the cell wall composition of seven genotypes of *M. sinensis* was evaluated. The contents of cellulose, hemicelluloses, and lignin (Supplementary Fig. S1 at *JXB* online) were found to correspond to those described in the literature (Zhao *et al.*, 2014). Furthermore, significant differences were also found in the H:P ratio in cell walls (Supplementary Fig. S2). The three genotypes that have lower contents of lignin (14–15.5%) and contrasting H:P ratios were chosen for a more in-depth evaluation of cell wall components, fine structure of cell wall polysaccharides, and saccharification efficiency. Cell walls were fractionated and the fractions were evaluated by monosaccharide analysis, oligosaccharide profiling, and glycome profiling using monoclonal antibodies. The different techniques used in this work showed different and complementary features of the cell wall fine structure of *M. sinensis* that were integrated in order to characterize some aspects of the composition and architecture related to saccharification efficiency and to recalcitrance.

### Fine structural features of *Miscanthus sinensis* cell walls

The results showed that the fine structure of arabinoxylan, the main hemicellulose of *Miscanthus* (Table 1; Le Ngoc Huyen *et al.*, 2010; Lygin *et al.*, 2011), is very similar in the three genotypes analysed, with a series of arabinoxylan oligosaccharides and a similar proportion of groups of oligosaccharides containing arabinose side chain decorations (Supplementary Fig. S3 at *JXB* online). Although there are similarities in arabinoxylan oligosaccharide profiles among genotypes, a significant difference in the xylan-5 group of antibodies (Fig. 4) was found in the sodium chlorite extract between H0120/H0116 and H0198. Similarly to sugarcane, which showed no or very low cross-reaction with xylan-5 antibodies in the sodium chlorite extract (De Souza *et al.*, 2013), H0120 and H0116 did not show any binding to this group of antibodies. The xylan-5 group of antibodies recognizes a 4-*O*-methyl GlcA side chain epitope in the context of an otherwise unmodified xylan backbone of at least four xylosyl residues (R. Clay, U. Avci, S. Pattathil. and M.G. Hahn, unpublished results). The presence of acetyl substituents on the xylan backbone blocks binding of this clade of antibodies (U. Avci and M.G. Hahn, unpublished data). Therefore, it is possible that the arabinoxylan residues present in the sodium chlorite extract of H0120 and H0116 are more acetylated than those present in H0198. Alternatively, it might be possible that the positioning of the arabinosyl branching could be different in H0198. These hypotheses will need confirmation with further and more in-depth analyses of polysaccharide fine structure.

According to the wall extract yield and monosaccharide results, arabinoxylan is present mainly in the 4M NaOH cell wall extracts (Fig. 2; Table 1). However, the endo-β-xylanase used for oligosaccharide profiling and the monoclonal antibodies used for glycome profiling were not effective at detecting these polymers in these fractions (Fig. 4; Supplementary Fig. S3 at *JXB* online), suggesting that the arabinoxylans present in the 4M NaOH extract probably differ considerably from the arabinoxylans extracted in the more soluble fractions. Although not detected by the endo-β-xylanase used, arabinoxylans are also present in the residue (Fig. 4; Table 1), suggesting that some of them may be strongly bound to cellulose microfibrils, as already proposed for maize and sugarcane (Kozlova *et al.*, 2014; Buckeridge *et al.*, 2015).

β-glucan, another major constituent of the cell wall of grasses, is present in several cell wall extracts of the three genotypes (Fig. 3), suggesting that this polysaccharide has different degrees of solubility in *M. sinensis* cell walls. Although Kiemle *et al.* (2014) demonstrated that β-glucan could interact directly with cellulose, the low amount of β-glucan in the 4M NaOH cell wall extracts of *M. sinensis* indicates that β-glucan is less strongly bound to the cellulose–arabinoxylan complex in this species. This result is similar to what has been observed for sugarcane (De Souza *et al.*, 2013).

It was also observed that β-glucan could not be detected by monoclonal antibodies in the glycome profile in either H0120 or H0116 (Fig. 4), although it could be detected by oligosaccharide profiling in most cell wall extracts in these two genotypes (Fig. 3). A possible explanation for this difference in detection might be related to the fact that the antibodies bind to epitopes by recognizing relatively larger regions of the main chain in comparison with the lichenase. The molecular weight of β-glucan in H0120 and H0116 could be lower than that in the H0198 genotype, so that the antibody was only able to bind in the β-glucan from the H0198 genotype. It is known that glycans must have a certain size to bind to the ELISA plates, but this hypothesis remains to be investigated.

As expected for the cell wall of grasses, xyloglucan is present in relatively small proportions in the cell walls of the three genotypes of *M. sinensis*. The oligosaccharide profiles show a higher amount of xyloglucan released from sodium chlorite and 0.1M NaOH extracts (Supplementary Fig. S4 at *JXB* online). This pattern is different from what was found in sugarcane stems, where xyloglucan oligosaccharides were released mainly in the 0.1M and 4M NaOH extracts (De Souza *et al.*, 2013). The presence of a higher amount of xyloglucan in the sodium chlorite extract suggests that at least these three analysed genotypes of *M. sinensis* have xyloglucan that might be branched with phenylpropanoids and/or lignin. Although the oligosaccharide profile showed the presence of xyloglucan in the sodium chlorite and 0.1M NaOH extracts, this polysaccharide was not recognized by the monoclonal antibodies (Fig. 4), which could indicate some differences in molecular weights in the same way that was explained above for β-glucans. On the other hand, xyloglucan was detected using the monoclonal antibodies in the oxalate extracts of the genotypes H0120 and H0116 (Fig. 4), but these observations were not confirmed when the oxalate extracts were treated with XEG (Supplementary Fig. S4), suggesting that although the xyloglucan polymer is present in these extracts, it probably displays a fine structure (e.g. branches with phenylpropanoids and/or with acetyl esters) that prevents attack of the enzyme on the main chain. Though in lower amount, some of the xyloglucans also seem to be more closely associated with cellulose as it is only extracted with higher concentrations of alkali (Fig. 4; Supplementary Fig. S3 at *JXB* online). This xyloglucan might be, as proposed by Buckeridge *et al*. (2005), one of the polymers tethering the cellulose microfibrils into cellulose macrofibrils. According to the present results, the fine structure of xyloglucans in *M. sinensis* appears to be quite complex, pointing to the possibility that there is some more soluble xyloglucan, along with xyloglucans more strongly bound to cellulose and to one another in the walls. Further studies capable of characterizing these xyloglucan classes structurally, as well as the manner of their interactions with monoclonal antibodies, are needed in order to clarify how xyloglucans participate in the wall assembly in *M. sinensis*.

Relatively little mannose was detected in the cell wall fractions of *M. sinensis* analysed (Table 1). In fact, mannans are minor components of cell walls of grasses (Vogel, 2008; Schröder *et al.*, 2009), and usually there is no binding with groups of antibodies related to (galacto-)mannans in this type of plant (De Souza *et al.*, 2013; DeMartini *et al*., 2013). However, epitopes for several monoclonal antibodies specific for the acetylated mannan and in the galactomannan-2 antibody groups cross-reacted exclusively with polymers present in cell wall extracts of the H0198 genotype (Fig. 4), suggesting that these polysaccharides are present in this genotype.

Most of the pectin polymers were released in the ammonium oxalate extract (Fig. 4). Considering the ammonium oxalate extract yield (Fig. 2) and also the monosaccharide composition (Table 1), pectin polysaccharides are present in the walls of *M. sinensis* in rather low proportions. However, the H0120 genotype showed a higher ammonium oxalate extract yield than the other two genotypes (Fig. 2). Similarly to sugarcane (De Souza *et al.*, 2013), homogalacturonan (HG), RG-I, and AGs are the main components found in *M. sinensis* (Fig. 4). The amount of arabinose detected in the ammonium oxalate extract (Table 1) suggests that some arabinan chains could be attached to RG-I.

The overall analysis of cell walls of *M. sinensis* indicates that although the walls of this plant are very similar to those of other grasses in terms of large classes of polymers (cellulose, hemicelluloses, pectins, and lignin), the fine structure probed with oligosaccharide and glycome profiles suggests a complexity that differs from that of other grass species. This hypothesis is corroborated by the fact that the comparisons made with sugarcane revealed that the two species seem to show some differences regarding the fine structures of arabinoxylans, xyloglucans, β-glucans, and pectins. Furthermore, the more in-depth analysis of the fine structure of *M. sinensis* cell walls demonstrates that subtle changes in epitopes appear to exist among genotypes. Thus, a question that arises from these observations is whether such fine structural differences would be involved on the liability of *M. sinensis* cell walls to hydrolysis aiming at bioenergy production.

### Influence of cell wall polysaccharide fine structure on saccharification efficiency and recalcitrance of *Miscanthus sinensis*

The comparison among three genotypes (H0120, H0116, and H0198) with different levels of lignin subjected to saccharification revealed that lignin can influence hydrolysis up to a certain level. For H0120 and H0116, saccharification efficiency was negatively correlated to lignin, whereas for H0198 no such correlation was observed at all (Fig. 6). Thus, the higher saccharification efficiency of H0198 can be thought of as being correlated to cell wall polysaccharides, with little or no influence of lignin. Although, the H0198 genotype contains ~3% more cellulose than the H0120 genotype (Fig. 1B), this does not explain the differences in saccharification efficiency. Indeed, the saccharification efficiency is 100% higher in H0198 compared with H0120 (Fig. 5A). 

This pattern made possible a more in-depth investigation of which polysaccharide features might be associated with higher saccharification efficiency in a biomass where lignin has low or no influence, namely the genotype H0198 in comparison with the other two genotypes. The top 50% variables in PC1 (Table 2A, 2B) showed that pectins are the most important variable, corresponding to 27.9% of all variables that favour saccharification of biomass of the H0198 genotype. These pectins are related to RG-I and AG polymers present specifically in the sodium chlorite, 0.1M NaOH, and 1M NaOH extracts (Table 2A). In switchgrass, the removal of enzyme-accessible pectins by treatment with endopolygalacturonase (EPG) and pectin-methylesterase (PME) did not influence the saccharification (De Martini *et al*., 2013). However, the RG-Is and AGs that are likely to influence saccharification efficiency in *M. sinensis* in the present study are released in the 0.1M and 1M NaOH extracts, suggesting that the major influence of pectins in saccharification efficiency is related to the pectic polymers more strongly bound to other cell wall components. Even when the influence of lignin content on saccharification efficiency is considered (i.e. analysing the principal components of H0120 and H0116), some epitopes in the polymer RG-I associated with the 1M NaOH extract seem to favour saccharification (Table 2C). Polymers released by sodium chlorite extraction are probably associated with lignin, since sodium chlorite is the fractionation step that leads to detachment of lignin from polysaccharides by breaking the ester linkages within the wall. Pectins containing AG epitopes in the sodium chlorite extract are the most abundant contributors to recalcitrance when lignin is highly correlated to saccharification efficiency (Table 2D), suggesting that these polymers might be associated with lignin in the *M. sinensis* cell walls, promoting a barrier to saccharification. Also in the cases where the lignin content has no correlation to saccharification efficiency (Table 2A), AG-1 as well as RG-I epitopes released in the sodium chlorite extract display a contribution to saccharification efficiency.

Although mannans appear to be present in very small proportions in the walls of *M. sinensis*, acetylated mannan and galactomannan-2 epitopes present in the ammonium oxalate-, sodium chlorite-, 0.1M NaOH-, and 1M NaOH-solubilized cell wall fractions explained 16% of the variation of PC1 that favoured saccharification in H0198. The presence of soluble β-glucans in the 0.1M and 1M NaOH extracts when the lignin content did not interfere in saccharification also contributed (~4%) to the saccharification efficiency (Table 2A). On the other hand, in genotypes where lignin influences the saccharification efficiency (e.g. in H0120 and H0116), β-glucans present in the 1M NaOH extract appear to be a variable that contributed to recalcitrance (Table 2D). Additionally, xylans in the sodium chlorite extract containing the xylan-5 epitope are also positively correlated to saccharification efficiency in H0198 (Table 2A). In cell wall fractions of poplar, De Martini *et al.* (2013) found that xylan-5 epitopes appear to be associated mostly with lignin and, apparently, they did not have any correlation to recalcitrance. In *M. sinensis*, the occurrence of xylan-5 epitopes in the sodium chlorite extract in H0198 also did not interfere with recalcitrance, even though this genotype has the highest lignin content among the analysed genotypes. In contrast, xylan-5 epitopes in *M. sinensis* are positively correlated to saccharification.

Xyloglucans and xylans present in the ammonium oxalate and 4M NaOH extracts seem to contribute with ~26% and ~13%, respectively, to cell wall recalcitrance when lignin is not correlated to saccharification (Table 2B). These two polymers have been hypothesized as playing a role in tethering cellulose microfibrils in grasses (Carpita and Gibeaut, 1993; De Souza *et al.*, 2013; Kozlova *et al.*, 2014). Also, the presence of these two types of polysaccharides in the 4M NaOH extract could be related to the assembly of cellulose microfibrils in the form of agglomerates (the macrofibrils) as already proposed for maize and sugarcane by Ding and Himmel (2006) and Buckeridge *et al.* (2015), respectively. Both can influence the recalcitrance since the polysaccharide interaction could hide the binding sites, displaying a fine structure inaccessible to the enzymes present in the cocktails used for saccharification.

However, when lignin is correlated to saccharification efficiency, both xylans and xyloglucans are not correlated to recalcitrance (Table 2C). Indeed, these are the main polysaccharides that favour saccharification efficiency. This result could be related to the fact that when lignin interferes with saccharification efficiency in *M. sinensis*, it seems to be due the arrangement of this polymer with pectins as discussed above. It is notable that when lignin is present, many epitopes related to pectins (specially AG epitopes) co-occur among the variables that favour recalcitrance (Table 2D), suggesting that both of these polymers are cross-linked, as already proposed in the literature (Monro *et al.*, 1972; Fry, 1983; De Martini *et al.*, 2014).

## Conclusions

Both lignin and polysaccharides can influence hydrolysis and recalcitrance of cell walls of *M. sinensis*. There seem to exist different patterns of polysaccharide epitopes and distinct oligosaccharides among genotypes that favour or do not favour cell wall hydrolysis. In *M. sinensis*, when lignin does not correlate with saccharification, pectins and mannans appear to favour hydrolysis, whereas recalcitrance can be explained by certain xyloglucans and arabinoxylans. When lignin is negatively correlated with saccharification, different types of arabinoxylans, xyloglucans, and pectins contribute to hydrolysis, whereas other types of pectins, probably associated with lignin, contribute to recalcitrance. Thus, the present results suggest that the manner in which lignin is linked to polysaccharides and the polysaccharide–polysaccharide interactions within cell walls can substantially change the way in which cell walls respond to attack by degradative enzyme cocktails.

## Supplementary data

Supplementary data are available at *JXB* online.

Figure S1. Percentage of lignin, hemicelluloses, and cellulose of seven genotypes of *Miscanthus sinensis*.

Figure S2. Hexose:pentose ratio from monosaccharide analysis of different genotypes of *Miscanthus sinensis*.

Figure S3. Oligosaccharide profiles obtained using xylanase (for detection of arabinoxylan) of cell wall extracts of stems of three different genotypes of *Miscanthus sinensis*.

Figure S4. Oligosaccharide profiles obtained using xyloglucan endoglucanase (for detection of xyloglucan) of cell wall extracts of stems of three different genotypes of *Miscanthus sinensis*.

Table S1. Monosaccharides (%) of cell wall extractions from the AIR (alcohol-insoluble residue) of seven genotypes of *Miscanthus sinensis*.

Table S2. Listing of plant cell wall monoclonal antibodies used for glycome profiling analyses.

Table S3. (A) Eigenvalues and proportions of each principal component generated by PCA using the cell wall variables from three genotypes. (B) PCA loadings calculated for each cell wall variable.

Table S4. (A) Eigenvalues and proportions of each principal component generated by PCA using the cell wall variables from genotypes H0120 and H0116. (B) PCA loadings calculated for each cell wall variable.

Supplementary Data
