# Supplementary material for: How cell wall complexity influences saccharification efficiency in Miscanthus sinensis
Source: J Exp Bot. 2015 Apr 23;66(14):4351–65. doi: 10.1093/jxb/erv183 (PMC4493786; doi:10.1093/jxb/erv183)
Supplement: Supplementary Data [file supp_66_14_4351__index.html]

How cell wall complexity influences saccharification efficiency in Miscanthus sinensis — How cell wall complexity influences saccharification efficiency in Miscanthus sinensis — Supplementary Data 

# How cell wall complexity influences saccharification efficiency in *Miscanthus sinensis*

## Supplementary Data

Data files

**Files in this Data Supplement:**

- Supplementary Data - Supplementary Data
- Supplementary Data - Supplementary Data
