# Supplementary material for: How cell wall complexity influences saccharification efficiency in Miscanthus sinensis
Source: J Exp Bot. 2015 Apr 23;66(14):4351–65. doi: 10.1093/jxb/erv183 (PMC4493786; doi:10.1093/jxb/erv183)
Supplement: Supplementary Data [file supp_erv183_jexbot141879_file001.pdf]

How cell wall complexity influences saccharification efficiency in Miscanthus sinensis

Amanda P De Souza, Claire L A Kamei, Andres F Torres, Sivakumar Pattathil, Michael G Hahn, Luisa M Trindade, and Marcos S Buckeridge

Supplemental Files

Supplementary Table S1. Monosaccharides (%) of cell wall extractions from AIR (alcohol insoluble residue) of seven genotypes of Miscanthus sinensis. Statistically significant differences are shown in bold (n=5).

Supplementary Figure S1. Percentage of (A) lignin, (B) hemicellulose, and (C) cellulose of seven genotypes of Miscanthus sinensis. Bars represent main values of technical replicates using a pool of biological replicates. The colored bars indicate the genotypes that were chosen for deeper cell wall analyses.

Supplementary Figure S2. Hexose:Pentose ratio (H:P) from monosaccharides analysis of different genotypes of Miscanthus sinensis. The colored bars indicate the genotypes that were chosen for deeper cell wall analyses.

Supplementary Figure S3. Oligosaccharide profiles obtained using xylanase (for detection of arabinoxylan) of cell wall fractions of stems of three different genotypes (H0120, H0116 and H0198) of Miscanthus sinensis. (a) - xylose, (b) - xylobiose, (c) –oligosaccharides containing no matching xylooligosaccharides homologous series (d) -- arabinosylated oligosaccharides. Missing chromatogram from ammonium oxalate fraction of H0116 genotype due to low amount of sample in this fraction. Data on chromatograms are averages of three biological replicates.

Supplementary Figure S4. Oligosaccharide profiles obtained using xyloglucan endo-glucanase (XEG, for detection of xyloglucan) of cell wall fractions of stems of three different genotypes (H0120, H0116 and H0198) of Miscanthus sinensis. Oligosaccharides from xyloglucan were not identified. Missing chromatogram from ammonium oxalate fraction of H0116 genotype due to low amount of sample in this fraction. Data on chromatograms are averages of three biological replicates.

Table S1.

Genotypes	Monosaccharides (%)					
	**Arabinose**	**Fucose**	**Galactose**	**Glucose**	**Rhamnose**	**Xylose**
H0227	4.89 ± 0.34	0.06 ± 0.02	1.37 ± 0.16	59.08 ± 1.10	0.03 ± 0.02	35.12 ± 0.85
H0120	6.09 ± 0.75	0.05 ± 0.02	1.58 ± 0.23	**54.44 ± 1.42**	n.d.	39.76 ± 3.62
H0119	5.15 ± 0.51	0.04 ± 0.02	1.72 ± 0.11	57.72 ± 0.43	0.02 ± 0.01	35.87 ± 1.49
H0116	4.93± 0.54	0.05 ± 0.01	1.35 ± 0.18	**63.72 ± 0.94**	0.02 ± 0.00	32.46 ± 0.42
H0117	6.02 ± 0.47	0.10 ± 0.02	1.78 ± 0.23	57.12 ± 0.80	**0.08 ± 0.01**	34.50 ± 0.78
H0198	3.84 ± 0.57	0.02 ± 0.01	1.00 ± 0.13	60.29 ± 1.56	0.04 ± 0.01	34.81 ± 1.09
H0201	4.95± 0.38	0.04 ± 0.01	1.40 ± 0.17	58.73 ± 0.92	0.06 ± 0.00	34.83 ± 1.16
P-value	**0.066***	0.441	**0.071***	**0.017**	**0.019**	0.128

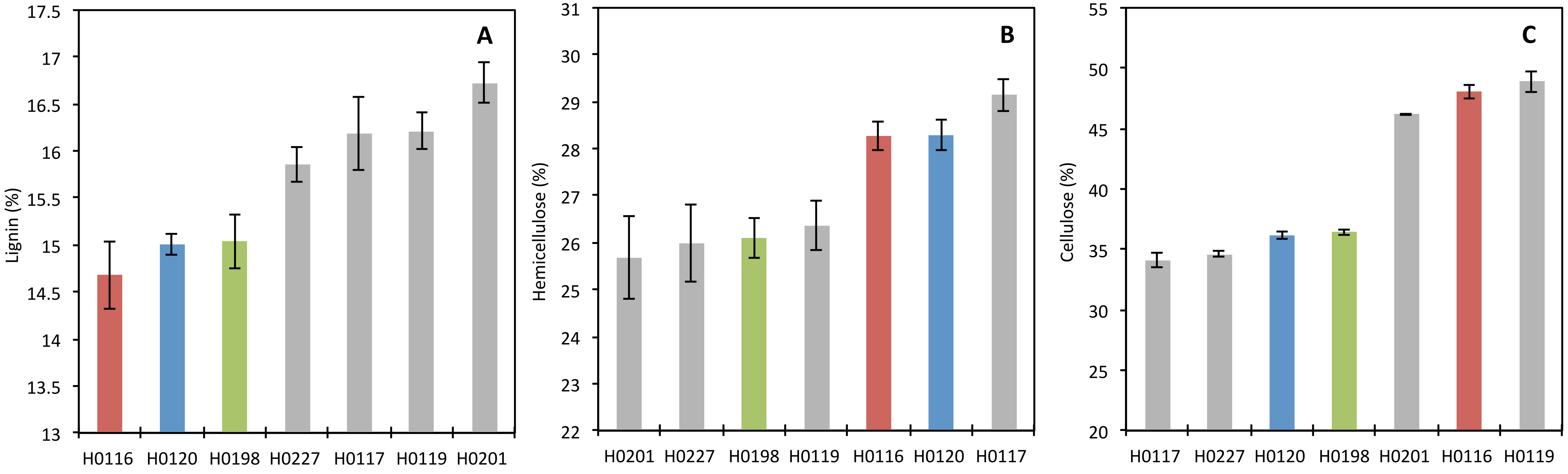

Supplemental Figure S1

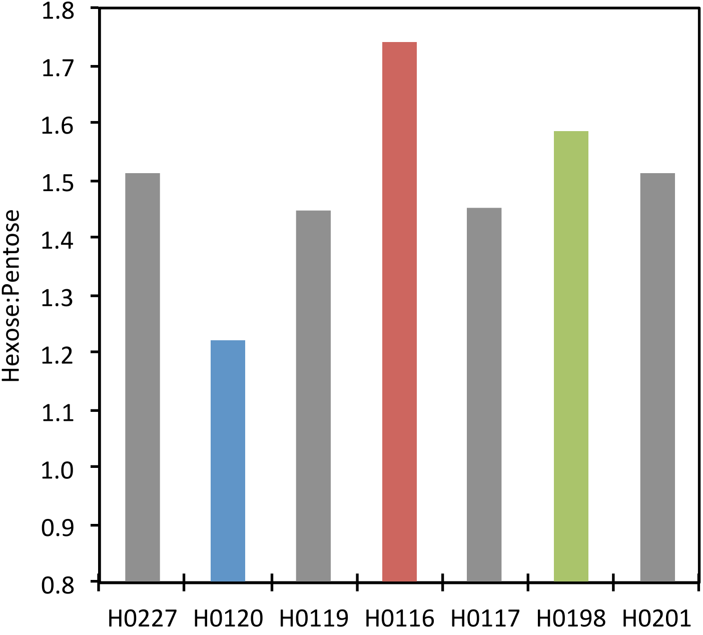

Supplemental Figure S2

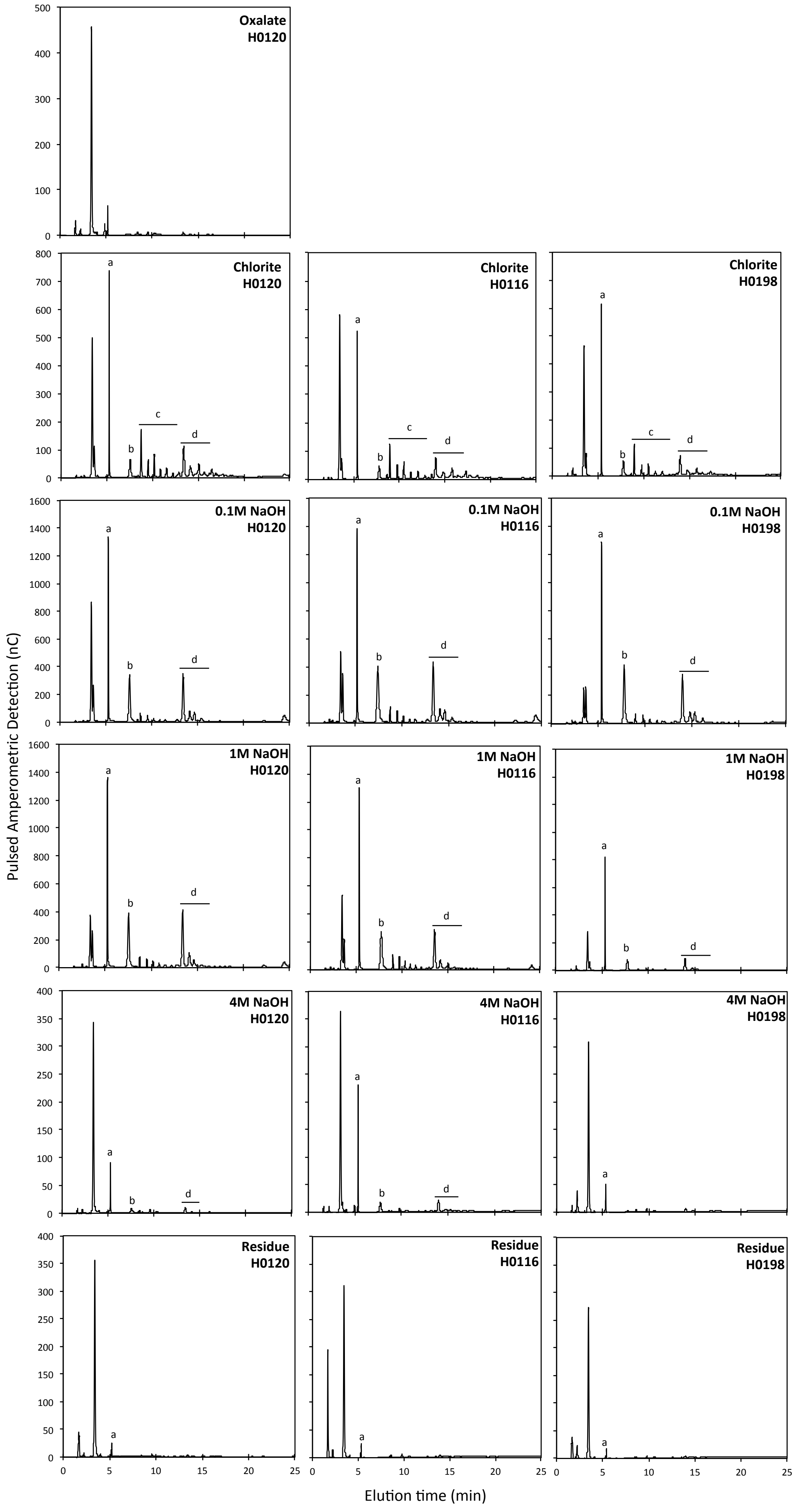

Supplemental Figure S3

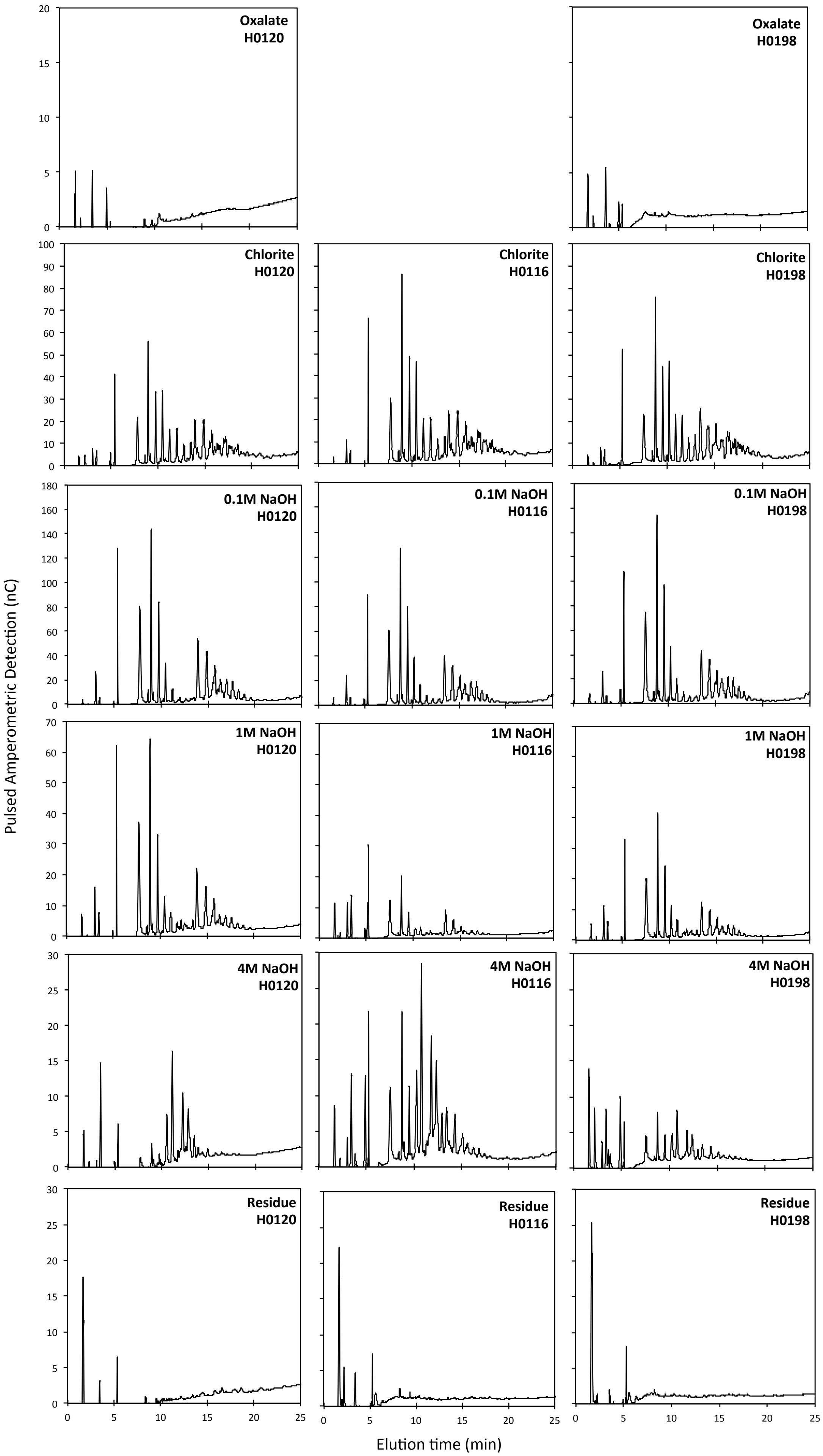

Supplemental Figure S4
